# Influence of the Initial Cell Number on the Growth Fitness of *Salmonella* Enteritidis in Raw and Pasteurized Liquid Whole Egg, Egg White, and Egg Yolk

**DOI:** 10.3390/foods10071621

**Published:** 2021-07-13

**Authors:** Silvia Guillén, María Marcén, Ignacio Álvarez, Pilar Mañas, Guillermo Cebrián

**Affiliations:** Departamento de Producción Animal y Ciencia de los Alimentos, Facultad de Veterinaria, Instituto, Agroalimentario de Aragón—IA2—(Universidad de Zaragoza-CITA), 50013 Zaragoza, Spain; silviaguillen@unizar.es (S.G.); mariamarcenterraza@gmail.com (M.M.); ialvalan@unizar.es (I.Á.); manas@unizar.es (P.M.)

**Keywords:** egg products, inoculum dose, growth rate, thermal treatments, foodborne pathogen

## Abstract

*Salmonella* growth in egg and egg products has been widely studied, but there are still some aspects that are not fully known. The objective of this work was to study the influence of the initial cell number on the growth fitness of *Salmonella* Enteritidis in raw and pasteurized egg products. Growth curves of five *Salmonella* Enteritidis strains in raw and pasteurized egg products, starting from different initial numbers, were obtained and fitted to the Baranyi and Roberts model. The results revealed that lower initial numbers led to longer lag phases (*λ*) and lower maximum specific growth rates (*μ_max_*) in raw liquid whole egg. Similar results were observed in raw egg white (except for one strain). Conversely, no influence (*p* > 0.05) of the initial concentration on *Salmonella* growth parameters in raw egg yolk was observed. On the other hand, no influence of the initial number of cells on *Salmonella* growth fitness in commercial pasteurized liquid whole egg was observed. The results obtained demonstrate that the disappearance of this initial-dose dependency phenomenon was dependent on the intensity of the thermal treatment applied. Finally, the influence of the initial number was, in general, lower in pasteurized than in raw egg white, but large differences among strains were observed.

## 1. Introduction

The microorganisms of the genus *Salmonella* are the second most commonly reported causative agent of foodborne outbreaks in the European Union [1] and constitute one of the major public health challenges worldwide. One of the most important sources of *Salmonella* contamination is eggs and egg products. Thus, raw and undercooked eggs are still the products most frequently identified as responsible for foodborne *Salmonella* infections in the European Union (37.0% of *Salmonella* outbreaks in Europe in 2019) [1].

Intact eggs can be contaminated by *Salmonella* using two possible routes, named vertical transmission and horizontal transmission. The first one (vertical transmission or the transovarian route) is due to the infection of the hen’s reproductive organs with *S.* Enteritidis and leads to the contamination of the yolk and/or the albumen during egg formation, i.e., before oviposition. The second possible route (horizontal transmission) is by penetration through the eggshell from the colonized gut or from contaminated feces during or after oviposition [2,3,4,5,6]. 

*Salmonella* growth and survival in egg and egg products has been widely studied, in particular, that of the serovar Enteritidis, because it is the predominant serovar in foodborne diseases associated with the consumption of these products [7,8,9]. It is well-known that there are wide differences in *Salmonella* growth ability depending on the egg fraction (yolk vs. white), growth temperature, and other factors [10,11,12]. *Salmonella* growth fitness is much greater in egg yolk than in the egg white due to the particular composition and physico-chemical characteristic of the later, which is particularly efficient in the inhibition on microbial growth of microorganisms. Thus, egg white has a high viscosity, an alkaline pH, and a number of antimicrobial components, including lysozyme, ovotransferrin, and several vitamin chelating proteins [13,14]. On the other hand, the influence of growth temperature on *Salmonella* growth in egg yolk and white as well as in liquid whole egg has been thoroughly studied, and there are many works dealing with this topic. Growth models have been developed in order to predict the growth of *Salmonella* in egg products in an effort to establish the optimal temperature and time for their preservation and distribution and, therefore, to ensure their safety through appropriate quantitative microbial risk assessments [11,15,16]. 

Nevertheless, there are some aspects, such as the influence of the initial cell number and/or the physiological state of cells on *Salmonella* growth fitness (as well as in resistance to stress), that are still not fully known. Indeed, these factors have been highlighted as future research needs in order to improve current *Salmonella* growth models [17]. In addition, in some recent works, it has been reported that *Salmonella* growth in raw egg is slightly slowed down in comparison with pasteurized egg [9,18]. A similar finding was recently reported by Kang and co-workers when studying *Salmonella* growth kinetics in raw and pasteurized egg white [19]. This is a phenomenon of the highest relevance since it might imply a potential food safety risk if data obtained in raw products are extrapolated to heat-treated ones. Unfortunately, data regarding this particular phenomenon are still scarce, and the underlying mechanisms remain to be elucidated. 

Therefore, the aim of this work was to study the influence of the initial cell number on the growth fitness of *S.* Enteritidis in raw and pasteurized liquid whole egg, egg white, and egg yolk.

## 2. Materials and Methods

### 2.1. Bacterial Strains and Culture Conditions

Five strains belonging to *Salmonella enterica* serovar Enteritidis were used in this study. The strains of *S*. Enteritidis (STCC 4300, STCC 4155, STCC 4396, STCC 7160, and STCC 7236) were supplied by the Spanish Type Culture Collection. Cultures were grown in tryptic soy broth (Oxoid, Basingstoke, UK) supplemented with 0.6% *w*/*v* yeast extract (Oxoid, TSB-YE) in 96-well microtiter plates and incubated at 37 °C under static conditions, as described by Guillén et al. [20].

### 2.2. Growth Curves

Growth experiments were carried out in raw liquid whole egg, egg white, and yolk obtained from medium-sized raw eggs (53–63 g) purchased from a local supermarket and in commercial pasteurized liquid whole egg, egg white, and egg yolk (Pascual, Aranda de Duero, Spain). For some experiments (see below), raw liquid whole egg was exposed to heat treatments simulating pasteurization conditions. Absence (25 g) of *Salmonella* in each batch of eggs and/or egg products used was checked following standard protocols (ISO 6579-1:2017).

The different egg products were inoculated with different initial doses (between 10^2^ (low dose) and 10^6^ (high dose) CFU/mL) of *Salmonella,* and were then incubated at 37 °C. Samples were taken at preset time intervals from 0 to 30 h, adequately diluted in buffered peptone water (Oxoid, BPW), and plated in Xylose Lysine Deoxycholate agar (Oxoid, XLD), which was used as the recovery medium. XLD plates were incubated for 48 h at 37 °C, and the number of colony-forming units (CFU) per plate was counted.

### 2.3. Thermal Treatments

Thermal treatments (the pasteurization of raw liquid whole egg) were carried out in a specially designed thermoresistometer implemented with a compatible control thermostat that allowed the performance of heating ramps at different rates [21]. Briefly, this instrument consists of a 400 mL vessel provided with an electrical heater for thermostation, a cooling system, an agitation device to ensure distribution and temperature homogeneity, and ports for the injection of microbial suspension and for the extraction of samples. The thermoresistometer was programmed to perform a linear temperature profile from 25 °C to the target temperature at a rate of 1 °C/min and held at that temperature (±0.1 °C). After treatments, pasteurized liquid whole egg was cooled and stored at 4 °C. The raw liquid whole egg was exposed to different treatment conditions simulating different pasteurization conditions: 60 °C for 3.5 min and 70 °C for 1.5 min.

### 2.4. Growth Curve Fitting and Statistical Analysis

Growth curves were constructed by plotting the logarithm of the number of *Salmonella* vs. time at the different condition assays. Each point of the growth curve corresponds to the average value of all the samples analyzed (at least three replicates). The curves obtained were fitted with the Baranyi and Roberts model (Equations (1) and (2)) [22]:

(1) $$Y_{t}=Y_{0}+\mu_{max}\cdot A_{t}-\frac{Y_{max}-Y_{0}}{M}\cdot ln\left[1-e^{-M}+\left(e^{-M}\cdot \frac{Y_{max}-Y_{0}-\mu_{max}\cdot A_{t}}{Y_{max}-Y_{0}}\right)\right]$$

(2) $$A_{t}=t-\lambda \cdot \left[1-\frac{1}{h_{0}}\cdot ln\left(1-e^{-h_{0}\cdot \frac{t}{\lambda }}+e^{-h_{0}\cdot \left(\frac{t}{\lambda }-1\right)}\right)\right]$$

where *Y_t_* is the Log_10_ of cell concentration at time *t* (CFU/mL), *Y*_0_ is the Log_10_ of the initial cell concentration (CFU/mL), *Y_max_* is the Log_10_ of maximum cell concentration (CFU/mL), *μ_max_* is the maximum growth rate (h^−1^), *λ* is the lag phase (h), and *M* and *h*_0_ are curvature parameters, taking them as constant values and with both set at a value of 10. For this purpose, GraphPad PRISM^®^ statistical software (GraphPad Prism version 8.00 for Windows, GraphPad Software, San Diego, CA, USA) was used. The same software was used for calculating the goodness of fit parameters (R^2^ and RMSE) and to carry out the statistical analysis (Student’s *t*-tests and ANOVA). Differences were considered significant for *p* ≤ 0.05.

## 3. Results

### 3.1. Influence of the Initial Concentration on the Growth Fitness of Salmonella Enteritidis STCC 4300 in Raw Liquid Whole Egg, Egg White, and Egg Yolk

**Figure 1:**
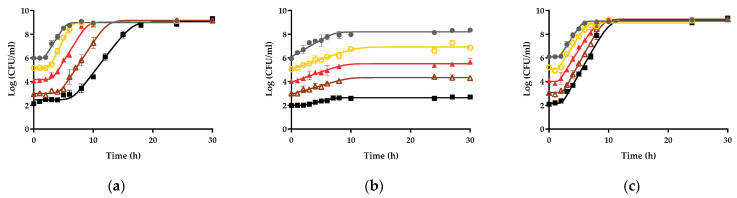
Effect of the inoculum dose on the growth fitness of Salmonella in raw liquid whole egg, egg white, and egg yolk: (**a**) Growth curves of *S*. Enteritidis STCC 4300 in raw liquid whole egg, (**b**) in raw egg white, and (**c**) in raw egg yolk. Initial dose 10^6^ CFU/mL ●, 10^5^ CFU/mL o, 10^4^ CFU/mL ▲, 10^3^ CFU/mL Δ, 10^2^ CFU/mL ∎. Lines correspond to the fit of the Baranyi model to the experimental data. Error bars represent the standard deviation.

First, the influence of the initial contamination dose on the growth fitness of *Salmonella* Enteritidis STCC 4300 (used as a model strain) in raw liquid whole egg, egg yolk, and egg white was determined. In order to do so, growth curves starting at different concentrations between 10^2^ (low dose) and 10^6^ (high dose) CFU/mL were obtained in the three egg products. As can be observed in Figure 1A and Table 1, the initial inoculum dose significantly affected the growth parameters calculated in raw liquid whole egg. The maximum growth rate in raw liquid whole egg determined for growth curves starting at a concentration of 10^2^ CFU/mL (0.663 ± 0.015 h^−1^) was significantly (*p* < 0.05) lower than curves starting at a concentration of 10^6^ CFU/mL (0.845 ± 0.038 h^−1^). Similarly, significant differences were also found among the lag values calculated in raw liquid whole egg. In this case, lag values increased as the dose inoculated in raw liquid whole egg decreased, with values of 6.32 ± 0.549 and 1.97 ± 0.038 h for the curves starting at 10^2^ and 10^6^ CFU/mL, respectively. A similar trend was observed in egg white, the egg product in which the lowest growth rates were obtained. Thus, in egg white, maximum growth rates of 0.164 ± 0.030 and 0.292 ± 0.066 h^−1^ were calculated from the growth curves corresponding to initial doses of 10^2^ and 10^6^ CFU/mL, respectively. The lag values in egg white also showed a similar trend to those obtained in raw liquid whole egg, with curves starting at 10^6^ CFU/mL not displaying any lag phase (not significantly different from 0; *p* > 0.05) and curves starting at 10^2^ CFU/mL showing the highest lag value (0.71 ± 0.193 h). It should be noted that in the particular case of raw egg white, the initial number also determined the growth yield (*Y_max_*). Furthermore, not only did it determine the growth yield (which was higher the higher the initial concentration) but also the difference between the initial and the final number of microorganisms (*Y_max_*–*Y*_0_), which was also higher the higher the initial number of microorganisms (1.18 Log_10_ cycles for growth curves starting at 10^6^ CFU/mL vs. 0.61 Log_10_ cycles for growth curves starting at 10^2^ CFU/mL). By contrast, no significant differences were found among the growth parameters calculated in raw egg yolk regardless of the inoculum dose. *S.* Enteritidis STCC 4300 maximum growth rates in egg yolk ranged from 0.837 ± 0.057 to 0.882 ± 0.047 h^−1^, corresponding to curves that started at 10^6^ and 10^3^ CFU/mL, respectively. Similarly, the lag value varied between 1.60 ± 0.140, for curves starting at 10^5^ CFU/mL, and 2.21 ± 0.240 h for curves starting at 10^3^ CFU/mL.

### 3.2. Growth Fitness of Salmonella Enteritidis STCC 4300 Cells in Raw and Pasteurized Egg Products

**Figure 2:**
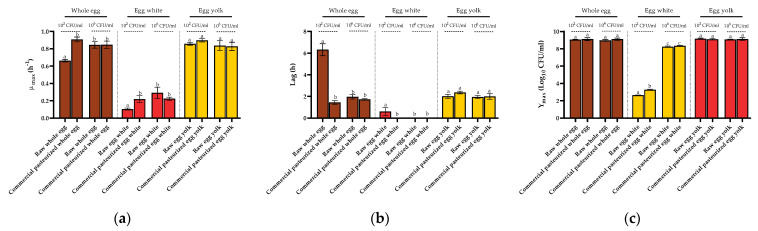
Growth parameters of *Salmonella* Enteritidis STCC 4300 cells in raw and commercial liquid whole egg, egg white, and egg yolk when inoculated at 10^2^ and 10^6^ CFU/mL. (**a**) *μ_max_* (h^−1^), (**b**) lag (h), and (**c**) *Y_max_* Log_10_ (CFU/mL) values calculated with the Baranyi model. Error bars represent the standard deviation, and letters indicate statistically significant differences.

Once the influence of the initial concentration on the growth fitness of *S.* Enteritidis STCC 4300 in raw egg products had been clearly established, the influence of the application of thermal (pasteurization) treatments to the three egg products on *Salmonella* growth fitness was studied. For this purpose, growth curves starting at 10^2^ (low dose) or 10^6^ CFU/mL (high dose) of *S.* Enteritidis STCC 4300 were obtained in commercial pasteurized liquid whole egg, egg white, and egg yolk, and compared with those obtained in raw (untreated) samples (Figure 2). 

As can be observed in Figure 2, when *S.* Enteritidis STCC 4300 cells were inoculated at the lowest concentration (10^2^ CFU/mL), their maximum growth rate in commercial pasteurized liquid whole egg was higher than that obtained in raw liquid whole egg at the same concentration, with values of 0.908 ± 0.028 and 0.663 ± 0.015 h^−1^, respectively. In parallel, the lag value also decreased considerably in commercial liquid whole egg, from 6.32 ± 0.549 h for raw liquid whole egg to 1.44 ± 0.178 h for commercial pasteurized egg. At these low inoculation doses, differences were also found between the estimated growth parameters in raw and commercial pasteurized egg white (Figure 2). Thus, the maximum growth rate in egg white was higher in commercial pasteurized egg white (0.219 ± 0.040 h^−1^) than in raw egg white, and no lag phase (not significantly different from 0; *p* > 0.05) was found in the former. In addition, significant differences in the *Y_max_* value (2.64 ± 0.037 vs. 3.28 ± 0.074 Log_10_ CFU/mL) for raw and commercial pasteurized egg white were also found. These results clearly indicate that prior application of thermal treatments to liquid whole egg and egg white would improve the growth fitness of the cells of this *Salmonella* strain in both egg products when inoculated with a low dose.

On the other hand, no significant differences (*p* > 0.05) were found in the growth parameters (*µ*_max_ and *λ*) of *S.* Enteritidis STCC 4300 obtained in commercial pasteurized liquid whole egg and egg white, regardless of the initial dose. Furthermore, growth parameters determined in commercial whole egg and egg white, regardless of the initial dose, were not significantly different (*p* > 0.05) to those determined in raw products when inoculated with 10^6^ CFU/mL, but were significantly different (*p* < 0.05) to those obtained in raw products inoculated with 10^2^ CFU/mL. Therefore, it can be concluded that pasteurization treatments would not influence the growth of *S.* Enteritidis STCC 4300 cells inoculated at the highest dose in these two egg products, and, what it is much more relevant, that the application of these commercial pasteurization treatments would abolish the initial-dose dependence of *S.* Enteritidis STCC 4300 growth parameters in liquid whole egg and egg white.

Finally, no significant differences (*p* > 0.05) were obtained in egg yolk regardless of the initial dose or thermal history (raw vs. commercial) of the egg product, which is consistent with the results indicated in the previous section.

**Figure 3:**
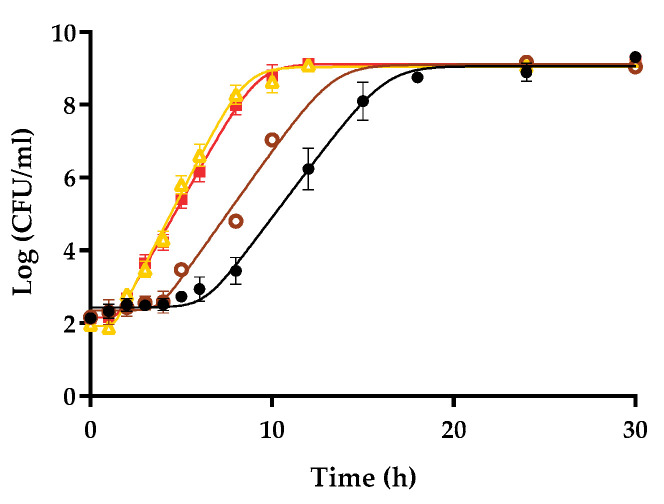
Influence of the intensity of pasteurization treatment on the growth fitness of *Salmonella* Enteritidis STCC 4300 cells inoculated at 10^2^ CFU/mL. Growth curves obtained in raw liquid whole egg (●), pasteurized whole egg at 60 °C 3.5 min (o), pasteurized whole egg at 70 °C 1.5 min (Δ), and commercial pasteurized liquid whole egg (■). Lines correspond to the fit of the Baranyi model to the experimental data. Error bars represent the standard deviation.

To further investigate the influence of heat treatment, raw liquid whole egg was exposed to thermal treatments of different intensity at different temperatures and times: 60 °C for 3.5 min and 70 °C for 1.5 min. Figure 3 shows the growth curves of *S.* Enteritidis STCC 4300 when inoculated with an initial dose of 10^2^ CFU/mL. Growth curves at the same initial dose in raw and commercial pasteurized liquid whole egg are also included for comparison purposes. As can be observed in the figure, the growth parameters calculated for *Salmonella* Enteritidis STCC 4300 cells in egg exposed to 60 °C for 3.5 min showed intermediate values between those calculated in commercial and raw liquid whole egg. In contrast, no significant differences (*p* < 0.05) were found between the growth parameters determined for *Salmonella* Enteritidis STCC 4300 cells in liquid whole egg pasteurized at 70 °C for 1.5 min and commercial pasteurized liquid whole egg. It was also found that when inoculated with high doses (10^6^ CFU/mL), no differences were found in the growth parameters determined, regardless of the thermal treatment, and that these values were not significantly different from those obtained in commercial liquid whole egg and liquid whole egg pasteurized at 70 °C for 1.5 min and inoculated with low doses (data not shown). In summary, it can be concluded that increasing the intensity of the thermal treatment led to a progressive increase in the growth rate and a decrease in the lag phase for *Salmonella* Enteritidis STCC 4300 inoculated with 10^2^ CFU/mL in liquid whole egg, and consequently, to the progressive disappearance of the initial-dose dependence of *S.* Enteritidis STCC 4300 growth fitness in this medium. 

### 3.3. Variability in Growth Fitness in Raw Liquid Whole Egg, Egg White, and Egg Yolk among S. Enteritidis Strains

In order to determine if the conclusions drawn above could be extrapolated to the whole *S.* Enteritidis serovar, the growth fitness of another four *S.* Enteritidis strains in the same media and conditions described above was studied. Therefore, growth curves of these four *S.* Enteritidis strains, starting at concentrations of 10^2^ and 10^6^ CFU/mL, were obtained in raw and commercial pasteurized liquid whole egg, egg yolk, and egg white.

**Figure 4:**
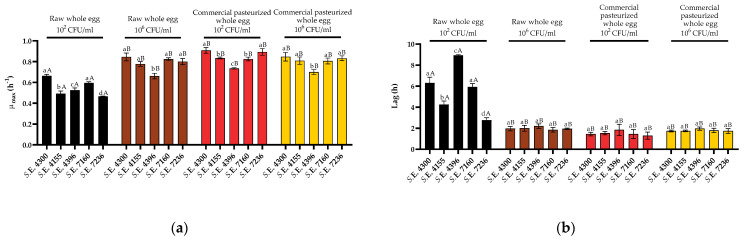
Growth parameters of five strains of *Salmonella* Enteritidis inoculated with 10^2^ and 10^6^ CFU/mL in raw liquid whole egg and commercial pasteurized liquid whole egg. (**a**) *μ_max_* (h^−1^) and (**b**) lag (h) values obtained after the fit of the growth curves to the Baranyi model. Error bars represent the standard deviation. Differences in the lower-case letters indicate statistically significant differences (*p* < 0.05) between strains grown on the same media and conditions (starting dose). Differences in the upper-case letters indicate statistically significant differences (*p* < 0.05) among growth conditions (raw vs. pasteurized and initial dose) for each strain.

Regarding liquid whole egg, the results obtained indicate that all the *S*. Enteritidis strains studied displayed a similar behavior to that described for strain STCC 4300 (Figure 4). Consequently, the lower growth rates (ranging from 0.456 ± 0.002 h^−1^ for *S.* Enteritidis STCC 4396, to 0.663 ± 0.015 h^−1^ for *S*. Enteritidis STCC 4300) and the highest lag values (ranging from 2.76 ± 0.244 h to 8.93 ± 0.080 h for *S.* Enteritidis STCC 7236 and *S.* Enteritidis STCC 4396, respectively) were observed in curves starting at 10^2^ CFU/mL in raw liquid whole egg. Additionally, as also described for strain STCC 4300, the highest growth rates and shorter lag times were observed, on average, in commercial liquid whole egg and raw egg when curves started at high concentrations (10^6^ CFU/mL). However, it should be noted that whereas for four of the five strains (STCC 4300, STCC 4155, STCC 7162, and STCC 7236), growth in raw liquid whole egg starting at low concentrations (10^2^ CFU/mL) resulted in significantly (*p* < 0.05) lower growth rates and longer lag times than in the other three conditions tested, with no significant differences (*p* > 0.05) in the growth parameters calculated among the later conditions, for strain STCC 4396, this lower growth fitness in raw liquid whole egg at low initial concentrations was mainly evidenced as an increase in the length of the lag phase. This latter strain, in contrast to the other four, also displayed a lower growth fitness in raw liquid whole egg than in pasteurized liquid whole egg, regardless of the initial dose. No significant differences were found in the *Y_max_* values regardless of the strain, initial dose, and/or heat treatment of the liquid whole egg studied (data not shown).

Overall, the differences in growth fitness among the different strains studied were higher in raw egg and, especially, at low inoculation doses. Thus, whereas significant differences were found among the growth rates and lag times of four out of five of the *S.* Enteritidis strains when they were grown in raw liquid whole egg inoculated with low doses (and more than a 3-fold difference in the lag times), only strain STCC 4396 displayed a significantly lower growth rate (only 15% lower than the average) in pasteurized liquid egg inoculated with high doses. In addition, it should be remarked that strain STCC 4396 was the one displaying the lowest growth fitness in all the conditions assayed.

**Figure 5:**
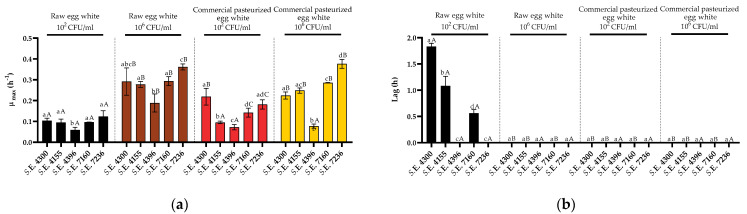
Growth parameters of five strains of *Salmonella* Enteritidis inoculated with 10^2^ and 10^6^ CFU/mL in raw egg white. (**a**) *μ_max_* (h^−1^) and (**b**) lag (h) values obtained after the fit of the growth curves to the Baranyi model. Error bars represent the standard deviation. Differences in the lower-case letters indicate statistically significant differences (*p* < 0.05) between strains grown on the same media and conditions (starting dose). Differences in the upper-case letters indicate statistically significant differences (*p* < 0.05) among growth conditions (raw vs. pasteurized and initial dose) for each strain.

Growth curves starting at high and low doses of the five *S*. Enteritidis strains were also obtained in raw egg white and commercial pasteurized egg white. As can be observed in Figure 5b, significant lag phases (significantly different from 0) were only observed for three strains and only when grown in raw egg white and starting from 10^2^ CFU/mL. As described for liquid whole egg, the lowest growth rates (on average) and highest lag phases were observed when cells were grown in raw egg white and starting from 10^2^ CFU/mL, and the highest growth rates were observed in egg white inoculated with 10^6^ CFU/mL (regardless of its prior thermal history) (Figure 5a). As also described for liquid whole egg, increasing the initial dose resulted in an increase in the growth rates in raw egg white; however, in this case, pasteurization did not abolish the initial-dose dependence of *Salmonella* growth. Thus, although the growth fitness of most *Salmonella* strains (3/5) starting at 10^2^ CFU/mL was higher in pasteurized than in raw egg white, it did not reach the values observed when 10^6^ CFU/mL was inoculated initially, i.e., the initial-dose dependence of *S*. Enteritidis growth was more relevant in egg white than in whole egg. 

*S*. Enteritidis STCC 4396 was once again the strain displaying the lowest maximum growth rates in all the conditions studied, with growth rates between 0.059 ± 0.013 h^−1^ and 0.188 ± 0.044 h^−1^. It also displayed significantly (*p* < 0.05) lower *Y_max_* values than the other four strains in three out of four of the conditions studied (data not shown). It should also be noted that, conversely to that observed in liquid whole egg, the variability in growth fitness (growth rates) among strains was higher in commercial than in raw egg white. 

**Figure 6:**
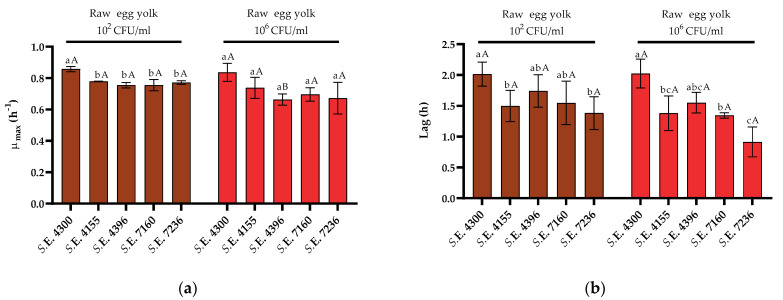
Growth parameters of five strains of *Salmonella* Enteritidis inoculated with 10^2^ and 10^6^ CFU/mL in raw egg yolk. (**a**) *μ_max_* (h^−1^) and (**b**) lag (h) values obtained after the fit of the growth curves to the Baranyi model. Error bars represent the standard deviation. Differences in the lower-case letters indicate statistically significant differences (*p* < 0.05) between strains grown on the same media and conditions (starting dose). Differences in the upper-case letters indicate statistically significant differences (*p* < 0.05) among growth conditions (raw vs. pasteurized and initial dose) for each strain.

Finally, as can be seen in Figure 6, the behavior of all the strains in raw egg yolk was similar to that of *S*. Enteritidis STCC 4300, with no changes in growth fitness regardless of the initial dose. It was also found that these parameters did not significantly change in pasteurized egg yolk (data not shown). In addition, it should be noted that the variability among the growth parameters calculated for *S.* Enteritidis strains in egg yolk was lower than that obtained in whole egg and egg white.

## 4. Discussion

The results presented in this paper demonstrate that the growth fitness of *Salmonella* Enteritidis cells in liquid whole egg and its fractions depends on the egg product (whole egg vs. white vs. yolk), its thermal history (except for egg yolk), the strain studied, and, in the case of raw liquid whole egg and egg white (both raw and pasteurized), the initial concentration of *Salmonella* cells. 

As expected, the lowest *S.* Enteritidis growth rates were observed in egg white and the highest in egg yolk. Nevertheless, in raw liquid whole egg at high initial concentrations and pasteurized (70 °C for 1.5 min) liquid whole egg (regardless of the initial concentration in the latter), the growth rates of most (4/5) *Salmonella* strains studied here were comparable to those in egg yolk. These differences in microbial growth fitness depending on the egg fraction studied have already been demonstrated by numerous authors, such as Kang et al., who reported that *Salmonella* growth in egg white was slower than that in liquid egg yolk and liquid whole egg [23], and Kim et al., who demonstrated that there is a difference in *Salmonella* growth fitness in unpasteurized liquid eggs depending on the type of liquid egg products (liquid whole egg, egg yolk, or egg white) and storage temperature [11]. As indicated in the introduction, *Salmonella* growth fitness is much greater in egg yolk than in egg white because the latter has a high viscosity, an alkaline pH, and a number of antimicrobial components, including lysozyme, ovotransferrin, and several vitamin chelating proteins [13,14]. Since liquid whole egg is a mix of both fractions, one would expect that *Salmonella* cells would display intermediate growth fitness, as reported here. 

In contrast, the influence of the initial dose on microbial (and particularly *Salmonella*) growth fitness has hardly been studied. Our results demonstrate that the initial number of cells can highly influence the growth fitness of *S.* Enteritidis cells in egg white and also in raw liquid whole egg. In this sense, it should be noted that Zaher and Fujikawa also observed a similar initial-dose effect phenomenon on *Salmonella* growth in raw ground chicken [24]. These authors attributed their results to the competition between *Salmonella* and natural microbiota, but this would likely not be the case for raw egg given the extremely low concentrations of microorganisms in this latter product. Regarding egg products, Kang et al. already determined that the bactericidal activity of egg albumen was dependent on the initial bacterial concentration. Thus, at low initial cellular concentrations, the antimicrobial factors of the egg albumen would be capable of inhibiting the growth of *S*. Enteritidis cells but would fail to control them when the bacterial concentration was higher [25]. Furthermore, in another study, it was reported that low increases in inoculum size, from 2 to 250 cells per egg, had a high impact on the ability of *S*. Enteritidis to migrate from egg white to yolk [26]. The authors established 250 cells as a critical level for *Salmonella* growth in the albumen and indicated that iron was the growth-limiting factor. In this sense, they suggested that over this threshold, *Salmonella* cells would be able to grow because they would be able to synthesize enough enterochelin, a bacterial siderophore that is able to compete with ovotransferrin for iron, or because the death of some cells in the albumen would allow the others to use them as a source of iron and/or energy [26].

Our results are consistent with these findings, although they show that *S.* Enteritidis cells would be able to grow (though very slowly and to very low yields) at concentrations as low as 10^2^ CFU/mL in egg white (slightly below the 250 limit previously determined). They also show that this initial-dose dependence would not be found in egg yolk and that, again, *Salmonella* cells would display an intermediate behavior in liquid whole egg. Thus, in raw liquid whole egg, *S.* Enteritidis maximum growth rates and lag times, but not the yields (final number of cells), would be initial-dose dependent; while this dose dependency is maintained in egg white upon its pasteurization, it will be abolished if the heat treatment applied to liquid whole egg is intense enough, as described below. These results can also be explained on the basis of the hypotheses already proposed [26], since this initial-dose dependence would be abolished (at least in liquid whole egg) upon the addition of iron to the medium [18]. Further work would be required in order to determine the growth fitness of *Salmonellae* in egg and egg products at lower initial doses than those studied here, since that might be the case in many cases/scenarios. This would be of the highest interest for the future development of improved growth models and risk assessments. 

The results reported here also demonstrate that *Salmonella* growth fitness in some egg products and conditions would also depend on the thermal history of those egg products. Thus, the application of thermal treatments on egg yolk would not impact *Salmonella* growth fitness regardless of the initial dose, likely because the growth rates in the “least favorable conditions” (i.e., raw product and low initial dose) would already be the highest achievable at this temperature for *Salmonella* cells as they were comparable to those obtained for these strains under the same incubation conditions in rich media, such as TSB-YE [27] (Guillén et al., 2021, submitted for publication). On the other hand, the application of thermal treatments on liquid whole egg led to an increase in *Salmonella* fitness in this product, but only when the initial number of cells was very low. Furthermore, it was demonstrated that this effect was higher the more intense the thermal treatments applied. This is consistent with the results of Sakha et al., who reported that a *S*. Enteritidis cocktail proliferated more rapidly in pasteurized liquid whole egg than in unpasteurized liquid whole egg, although it should be noted that they did not observe any influence of the initial dose on the growth parameters [9]. The lack of influence of thermal treatments on *Salmonella* growth fitness at high concentrations can be explained on the same basis as that of egg yolk. Thus, the growth rate of these *Salmonella* strains at high initial concentrations in this product is comparable to that in egg yolk and also in rich laboratory media. Finally, application of thermal treatments to egg white also resulted in an increase in growth fitness for *Salmonella* when inoculated at low concentrations. Nevertheless, in this case, and even after the application of the harsher treatment conditions tested (up to 120 min at 56.0 °C; data not shown), the growth rate in pasteurized egg white of cells inoculated at 10^2^ CFU/mL did not reach that of samples inoculated with 10^6^ CFU/mL. Similar results to those obtained here were obtained by Kang et al., who observed that the maximum growth rate of a cocktail of four *Salmonella* strains inoculated in pasteurized egg white at a dose of 3.5 ± 0.5 Log_10_ CFU/mL was much higher than in raw egg white [19]. 

Classical pasteurization treatments (1–10 min at 60–72 °C) applied in the industry to pasteurize whole egg are limited because of the sensitivity of the egg white proteins to heat treatments, which might lead to egg coagulation. Furthermore, pasteurization conditions should be even milder in egg white (<60 °C). Therefore, it can be speculated that the differences in growth rates between raw and pasteurized liquid whole egg and egg white would be due to the thermal denaturation of egg white proteins with antimicrobial properties, such as ovotransferrin and lysozyme [14,28]. Furthermore, since the concentration of these antimicrobial proteins would be lower in liquid whole egg than in egg white, this would explain why egg pasteurization treatments would be able to completely abolish the initial-dose dependence of *Salmonella* growth fitness in whole egg but not in egg white. Other causes that might also contribute to explaining this different behavior between egg white and whole liquid egg include the lower content of iron and other nutrients in egg white, its particular physical–chemical characteristics, and the lower intensity that can be applied in pasteurization treatments to egg white. Nonetheless, further work will be required to fully elucidate all the components of the egg with antimicrobial activity, including those limiting iron bioavailability, as indicated by Guillén et al. [18]. In this sense, recent studies also suggest that low-weight components (<10 kDa) of egg white are largely responsible for the bactericidal activity of egg white at high treatment temperatures [29]. 

Regarding the variability in growth fitness among strains, it should be noted that *S.* Enteritidis displays a higher survival capacity in egg white than other species, higher than other *Salmonella* serovars [30]. Nevertheless, it has also been reported that *S.* Enteritidis strains vary in their ability to grow or survive in egg white, indicating that some variants are better adapted to egg white [31,32,33]. Nonetheless, this advantage over other serovars in the growth and survival ability of *S.* Enteritidis in egg white only existed when the inoculum size was below 10^7^ CFU/mL [34]. Previous studies have reported that genes involved in iron acquisition, cell envelope structure, osmotic and oxidative protection, amino acid and carbohydrate metabolism, motility, and stress responses may contribute to the survival of *S.* Enteritidis in egg albumen [31,33,34,35]. Egg white is a very unfavorable medium for bacteria, and the major factor limiting bacterial growth in it is iron restriction, having a concentration of iron between 3.6 and 18 µM [36]. *Salmonella* has the ability to produce siderophores in iron-restricted media to cope with this restriction, as in egg white, where iron is assumed to be chelated by ovotransferrin [37]. However, little is known regarding the variability in the ability to synthesize these siderophores among *Salmonella* strains. In any case, it can be speculated that the differences in growth fitness observed among the different strains might be associated with a different siderophore-production ability. This will explain why little or no differences among strains were found in egg yolk and pasteurized liquid whole egg, whereas the highest ones were observed in egg white. Further work will be required in order to verify this hypothesis.

The risk of suffering salmonellosis is related to one or several of the following scenarios: the existence of a very high population of *Salmonella* in raw egg prior to pasteurization, the application of inadequate/insufficient treatment, or post-pasteurization contamination. The potential impact of post-processing contamination of liquid whole egg, egg white, and egg yolk was the one assessed in this study. According to our results, post-pasteurization recontamination of pasteurized liquid whole egg and egg white would represent a greater risk for consumers than that of the unpasteurized products. This is of the highest relevance since commercially pasteurized egg products are often used as ingredients in foods without any further heat treatment during food preparation. Furthermore, if only the risk associated with a potential recontamination is considered, the data obtained here indicate that the lowest intensity pasteurization treatments (such as 60 °C 3.5 min for liquid whole egg) would be more recommendable. It is noteworthy that low-intensity pasteurization treatments have been proposed to increase the digestibility and reduce the allergenicity of whole egg proteins [28]. 

Finally, it should also be noted that it is generally acknowledged that the initial dose does not determine the microbial maximum growth rates, which will only be determined by the microbial genotype and the growth conditions (intrinsic and extrinsic factors). However, although our results seem to contradict this affirmation, this is not the case. Thus, this general rule is based upon the assumption that the concentration (or bioavailability) of the limiting nutrient/s in batch cultures is fixed, but this is not the case here since the synthesis of siderophores and/or cell death (the two theories proposed for explaining the results obtained here) would increase the amount of iron and energy available in a dose-dependent manner.

## 5. Conclusions

In summary, our results demonstrate that the initial dose and thermal history of liquid whole egg and egg white can determine the growth fitness of *Salmonella* Enteritidis cells in these products, whereas this does not occur in egg yolk. In addition, our results also indicate that the variability in growth fitness among strains highly depends on the conditions in which it is studied. Further work will be required in order to fully elucidate the mechanisms underlying the results obtained here—particularly how the initial dose determines *Salmonella* growth fitness in some egg products/fractions and why pasteurized egg products provide more favorable conditions to *Salmonella* cells—and to develop novel predictive growth models and perform improved risk assessments of *Salmonella* in egg products, including all the factors that have been proven to affect its growth fitness in them. 

## Figures and Tables

**Table 1:** Growth (*Y*_0_, *Y_max_*, *λ*, and *μ_max_*) and goodness of fit (R^2^, RMSE) parameters (Baranyi and Roberts model) calculated for *S.* Enteritidis STCC 4300 in raw liquid whole egg, egg white, and egg yolk at 37 °C. Values presented correspond to the mean and standard deviations (SD, in parentheses). Letters indicate statistically significant differences.

*Y*_0_ (CFU/mL)	*µ_max_* (h^−1^)	*λ* (h)	*Y_max_* (CFU/mL)	R^2^	RMSE
Raw whole egg					
6.00 (0.061)	0.845 (0.038) ^a^	1.97 (0.038) ^a^	8.96 (0.093)	0.99–1.00	0.115–0.166
5.15 (0.066)	1.056 (0.142) ^a,b^	2.71 (0.230) ^b^	8.99 (0.013)	0.99–1.00	0.127–0.201
4.21 (0.086)	0.982 (0.039) ^b^	3.49 (0.128) ^c^	9.01 (0.079)	0.99–0.99	0.287–0.316
3.01 (0.035)	0.836 (0.018) ^a^	4.54 (0.505) ^d^	9.16 (0.038)	0.99–1.00	0.133–0.284
2.22 (0.070)	0.663 (0.015) ^c^	6.32 (0.549) ^e^	9.05 (0.030)	0.99–1.00	0.225–0.274
Raw egg white					
6.06 (0.116)	0.292 (0.066) ^a^	- ^a^	8.24 (0.084)	0.97–0.99	0.131–0.172
5.03 (0.026)	0.193 (0.022) ^a,b^	0.29 (0.082) ^b^	6.97 (0.051)	0.96–0.97	0.172–0.187
4.02 (0.023)	0.205 (0.014) ^a^	0.43 (0.094) ^b^	5.45 (0.045)	0.96–0.98	0.097–0.144
2.98 (0.059)	0.158 (0.010) ^b^	0.40 (0.211) ^b^	4.42 (0.120)	0.98 -0.99	0.055–0.096
2.03 (0.013)	0.104 (0.012) ^c^	1.83 (0.106) ^c^	2.64 (0.037)	0.95–0.99	0.067–0.138
Raw egg yolk					
6.10 (0.028)	0.837 (0.057) ^a^	1.94 (0.147) ^a^	9.07 (0.038)	0.99–0.99	0.121–0.181
5.07 (0.073)	0.829 (0.027) ^a^	1.60 (0.140) ^a^	8.93 (0.139)	0.98–0.99	0.249–0.257
4.05 (0.381)	0.838 (0.018) ^a^	1.89 (0.403) ^a^	9.09 (0.071)	0.99–1.00	0.163–0.264
3.09 (0.067)	0.882 (0.047) ^a^	2.21 (0.240) ^a^	9.27 (0.106)	0.99–1.00	0.134–0.235
2.15 (0.081)	0.857 (0.016) ^a^	2.01 (0.195) ^a^	9.23 (0.056)	0.98–0.99	0.271–0.414

## Data Availability

The data presented in this study are available upon request from the corresponding author.
